# Current conditions of use of long‐term care insurance services for home‐based long‐term care recipients who need insulin therapy

**DOI:** 10.1111/ggi.14556

**Published:** 2023-02-11

**Authors:** Shinobu Watanabe, Tomoko Ito, Takehiro Sugiyama, Makiko Tomita, Shu Kobayashi, Nanako Tamiya

**Affiliations:** ^1^ Health Services Research and Development Center University of Tsukuba Tsukuba Ibaraki Japan; ^2^ Department of Nursing, School of Health Sciences Ibaraki Prefectural University of Health Sciences Ami‐machi Ibaraki Japan; ^3^ Department of Health Services Research, Institute of Medicine University of Tsukuba Tsukuba Ibaraki Japan; ^4^ Diabetes and Metabolism Information Center, Research Institute National Center for Global Health and Medicine Tokyo Japan; ^5^ Institute for Global Health Policy Research, Bureau of International Health Cooperation National Center for Global Health and Medicine Tokyo Japan; ^6^ Analytics and Innovation Department Research and Development Group, SMS Co., Ltd. Tokyo Japan

**Keywords:** community and family medicine, diabetes mellitus, insulin, long‐term care

## Introduction

Although insulin therapy is based primarily on self‐management, it becomes difficult to manage as individuals age and their physical and cognitive functions decline. Long‐term care insurance is required to manage insulin therapy when these individuals need long‐term care and social support.^1^, ^2^ Currently, in Japan, if an older person needing insulin therapy becomes unable to manage the therapy by themselves, family members are often expected to provide it. If support from family members is not available, services from the long‐term care insurance system may be available. These services include home‐visit nursing provided by medical professionals to older persons who need nursing care or assistance with insulin therapy, as well as home‐visit care by non‐medical professionals. To date, there have been no studies describing the types of care services used by long‐term care insurance users with diabetes.

Therefore, this study aims to clarify the current use of long‐term care insurance services by older persons who have diabetes and require long‐term home care to receive insulin therapy.

## Methods

In this study, we used anonymized data from “Kaipoke,” a management support platform for elderly care operators provided by SMS Co., Ltd (https://global.bm-sms.com/service/care/). Kaipoke has been introduced in many home‐care support offices in each prefecture, which has been spread almost  nationwide in the use of long‐term care services. The data used in this study were extracted from care plans created by care manager within Kaipoke. We extracted the latest data, from April 2011 to April 2019, on recipients who were 65 years of age or older and who had diabetes or used insulin. We then grouped them by whether they lived with their families. A simple tabulation was performed on the attributes and the number of care recipients.

## Results

A total of 6627 people were identified as having diabetes or using insulin, 641 (9.7%) of whom used insulin. Of the insulin users, 373 lived with their families, and 268 lived alone. Of these, 167 people (44.7%) who lived with their families and 120 (44.8%) who lived alone required long‐term care at level 3 or higher. Among older persons with dementia, namely, those with an independence level of IIa or higher, indicating that they need support with their activities of daily living, 233 (62.5%) lived with their families, and 184 (68.6%) lived alone. Furthermore, among those who lived alone and used home‐visit care, 119 (58.0%) did not use home‐visit nursing. Regarding the use of long‐term care insurance services for insulin users living alone, 103 (38.4%) received home‐visit nursing, 205 (76.5%) received home‐visit care, 128 (47.8%) commuted for care, and 13 (4.9%) underwent short‐term admissions (Table 1). In contrast, 79 (47.9%) of the 165 older people living alone requiring insulin therapy who did not use home‐visit nursing care had a level of independence in daily life of an older person with dementia of IIb or worse (data not shown).

**Table 1:** Characteristics of users of insulin therapy according to their living situation (*n* = 641)

		Living with family	Living alone
		*n* = 373(%)	*n* = 268(%)
Sex	Male	183 (49.1)	120 (44.8)
	Female	190 (50.9)	148 (55.2)
Age	65–74	82 (22.0)	81 (30.2)
	75–84	190 (50.9)	121 (45.1)
	85+	101 (27.1)	66 (24.6)
Level of long‐term care need	Care Level 1	91 (24.4)	81 (30.2)
	Care Level 2	115 (30.8)	67 (25.0)
	Care Level 3	77 (20.6)	56 (20.9)
	Care Level 4	55 (14.7)	46 (17.2)
	Care Level 5	35 (9.4)	18 (6.7)
Degree of independence in the daily life of an older person with disabilities	Independence	9 (2.4)	13 (4.9)
	J1	22 (5.9)	11 (4.1)
	J2	37 (9.9)	32 (11.9)
	A1	63 (16.9)	54 (20.1)
	A2	95 (25.5)	69 (25.7)
	B1	54 (14.5)	33 (12.3)
	B2	55 (14.7)	36 (13.4)
	C1	15 (4.0)	11 (4.1)
	C2	23 (6.2)	9 (3.4)
Degree of independence in the daily life of an older person with dementia	Independence	66 (17.7)	39 (14.6)
	I	74 (19.8)	45 (16.8)
	IIa	47 (12.6)	45 (16.8)
	IIb	69 (18.5)	69 (25.7)
	IIIa	59 (15.8)	42 (15.7)
	IIIb	22 (5.9)	6 (2.2)
	IV	29 (7.8)	19 (7.1)
	M	7 (1.9)	3 (1.1)
Home‐visit nursing (*n* = 193)^†^	Use	90 (24.1)	103 (38.4)
Home‐visit care (*n* = 345)^†^	Use	140 (37.5)	205 (76.5)
Commuting for care (*n* = 343)^†^	Use	215 (57.6)	128 (47.8)
Short‐term stay at a care facility (*n* = 74)^†^	Use	61 (16.4)	13 (4.9)

^†^ By multiple answers.

## Discussion

In this study, we used large‐scale data to clarify the use of long‐term care insurance services for older persons requiring insulin therapy at home. Based on the results, nearly half of those living alone had a long‐term care level of 3 or higher, and more than 60% had a cognitive decline. Of the individuals who used insulin, the number who received home‐visit care and the number who commuted for care were both higher than the number of those who received home‐visit nursing? Approximately half of the people living alone who did not use home‐visit nursing had a cognitive decline at a level that made drug management difficult.

When older people who cannot manage insulin injections live alone, there is a possibility that social support, including long‐term care insurance services, other than medical services, will be inadequate. Currently, in Japan, only family members and medical workers are allowed to administer insulin injections on behalf of patients. Although it is legal for care workers to watch over the self‐injection of insulin,^3^ a previous study^4^ reported that non‐medical care workers might be confused about managing insulin at home when older persons cannot manage it by themself. The survey results showed that just under 60% of those using home care did not use home nursing. It is unclear what kind of insulin management support the elderly who did not use this home nursing service received. They may have family living separately or friends supporting them, but this would be a topic for future research, as no data were available. In the future, it may be necessary to develop a system that allows community members, such as non‐medically qualified caregivers, family members, and friends, to assist insulin‐using older individuals in insulin management.

## Disclosure statement

This research was funded by SMS Co., Ltd, grant number CRE30018, at the University of Tsukuba. This study was conducted as a joint research program at the University of Tsukuba and SMS Co., Ltd.

MT was a researcher at the University of Tsukuba for joint research with SMS Co., Ltd. in the 2020 financial year (i.e., April 2020 to March 2021). The research project was supported by SMS Co., Ltd. MT and SK were employed by SMS Co., Ltd. TS was hired by the University of Tsukuba using funds from SMS Co., Ltd for joint research in the 2020 financial year. The funders had no role in the design of the study; in the analyses or interpretation of data; in the writing of the manuscript; or in the decision to publish the results.

The requirement of patient consent was waived as the data contain no individually identifying information.

## Data Availability

The data that support the findings of this study are available from SMS Co. Ltd. Restrictions apply to the availability of these data, which were used under license for this study. Data are available from the authors with the permission of SMS Co. Ltd.
